# Complementary Genomic and Functional Potential of *Bacillus subtilis* RKM-1 and *Bacillus licheniformis* RKM-2 for Organic Waste Bioconversion and Composting

**DOI:** 10.3390/genes17101222

**Published:** 2026-10-01

**Authors:** Tekebayeva Zhanar, Abilkhadirov Arman, Begakhmet Akbota, Kulzhanova Kamshat, Temirbekova Aliya, Nurbekova Zhadyrassyn, Aslan Temirkhanov, Bekshin Zhandarbek, Abzhalelov Akhan

**Affiliations:** 1 Republican Collection of Microorganisms, Astana 010000, Kazakhstan; zanartekebaeva@gmail.com (T.Z.); aabilhadirov@gmail.com (A.A.); begakhmet_am@enu.kz (B.A.); atemirbekova94@gmail.com (T.A.); zhadyrassyn.nurbekova@gmail.com (N.Z.); aszhte@gmail.com (A.T.); zmbekshin@gmail.com (B.Z.); ab.akhan1959@gmail.com (A.A.); 2 Department of Management and Engineering in the Field of Environmental Protection, L.N. Gumilyov Eurasian National University, Astana 010000, Kazakhstan; 3 Department of Biotechnology and Microbiology, L.N. Gumilyov Eurasian National University, Astana 010000, Kazakhstan

**Keywords:** *Bacillus subtilis*, *Bacillus licheniformis*, whole-genome sequencing, comparative genomics, nitrogen metabolism, carbohydrate metabolism, composting, organic waste, sewage sludge

## Abstract

Background: Efficient bioconversion of organic waste requires microorganisms with the potential to degrade complex organic compounds and transform biogenic elements. Methods: In this study, two thermophilic bacterial strains, RKM-1 and RKM-2, isolated from sewage sludge, were characterized using whole-genome sequencing and comparative genomics to establish their taxonomic positions and assess their functional potential. Results: RKM-1 and RKM-2 were identified as *Bacillus subtilis* and *Bacillus licheniformis*, respectively, with maximum ANI values of 98.76% and 99.76%. Comparative genomic analysis revealed differences in the functional potential of the two strains. RKM-1 exhibited genomic features associated with the transformation of biogenic elements and secondary metabolite biosynthesis, whereas RKM-2 showed greater genetic potential for the utilization and transformation of complex carbohydrates, supported by the presence of genes such as *amyA*, *bglC*, *celA*, *glcT*, *galE*, *galU*, and *glgABCD*. These differences indicate potentially complementary functional profiles of the two strains in the bioconversion of organic substrates. During sewage sludge composting, inoculation with both strains accelerated the onset of the thermophilic phase. *Bacillus subtilis* RKM-1 reached the highest compost temperature (60 °C), whereas *Bacillus licheniformis* RKM-2 most effectively promoted compost maturation, reducing the C:N ratio to 9:1 within 60 days. Conclusions: These findings demonstrate a strong relationship between the genomic characteristics and functional traits of the investigated strains and highlight the value of comparative genomics as a powerful tool for identifying promising thermophilic microorganisms for efficient bioconversion of organic wastes.

## 1. Introduction

Microorganisms are major biological drivers of global biogeochemical cycles and mediate the transformation of carbon, nitrogen, phosphorus, and other elements essential for the functioning of terrestrial ecosystems. Through organic matter mineralization, assimilation of inorganic compounds, degradation of plant-derived polymers, and recycling of elements within biological systems, soil microorganisms influence soil fertility, nutrient availability, plant productivity, and the stability of agroecosystems [1,2,3].

Members of the genus *Bacillus* represent an important component of soil microbial communities. These bacteria exhibit pronounced physiological and metabolic versatility, form resistant endospores, and can maintain viability under nutrient limitation, desiccation, and other adverse environmental conditions [4].

Members of the *B. subtilis* species complex and *B. licheniformis* are widely distributed in soils, composts, plant residues, and other organic substrates. They are capable of secreting amylases, proteases, cellulolytic enzymes, and other hydrolytic enzymes involved in the degradation of complex organic compounds [5]. *B. subtilis* is also widely regarded as a model Gram-positive bacterium characterized by an efficient protein secretion system, biofilm-forming capacity, and the ability to synthesize a broad range of secondary metabolites [6].

Bacteria of the genus *Bacillus* are of interest not only as decomposers of organic matter but also as potential components of microbial preparations for crop production because of their ability to improve nutrient availability and suppress phytopathogens [5,6,7,8]. One of the mechanisms underlying the biocontrol potential of *Bacillus* is the production of secondary metabolites. Members of the *B. subtilis* species complex can produce lipopeptides, polyketides, siderophores, and volatile organic compounds involved in microbial competition and the suppression of phytopathogenic microorganisms [9,10].

Nitrogen metabolism in *Bacillus* involves diverse systems for the transport, reduction, and assimilation of inorganic nitrogen compounds, the composition of which may vary considerably even among closely related members of the family Bacillaceae [11,12]. Certain *Bacillus* species also possess mechanisms involved in phosphorus solubilization and mineralization, including phosphatase-mediated transformation of organic phosphorus, which may enhance phosphorus bioavailability [13].

Carbon metabolism and organic matter degradation are associated with the activity of hydrolytic enzymes, including amylases, cellulases, β-glucosidases, and other glycoside hydrolases that facilitate the breakdown of complex carbohydrate substrates. Such systems are particularly relevant to microorganisms with potential applications in plant residue processing and composting, as the rate of organic substrate bioconversion is largely determined by the functional activity of the microbial community [14,15]. The application of microorganisms with relevant functional capabilities may accelerate the biodegradation of organic compounds and improve the properties of the resulting compost [16].

Advances in whole-genome sequencing technologies have substantially transformed approaches to bacterial systematics and the functional characterization of microbial strains. Whole-genome analysis enables the simultaneous assessment of assembly quality, genome organization, functional gene content, biosynthetic gene clusters, and evolutionary relationships that cannot be reliably resolved solely on the basis of phenotypic characteristics or 16S rRNA gene sequences [17,18]. Whole-genome data are particularly valuable for investigating closely related members of the *B. subtilis* species complex, for which individual conserved markers may provide insufficient taxonomic resolution. Current approaches to genome-based taxonomy integrate average nucleotide identity (ANI) and digital DNA–DNA hybridization (dDDH) with phylogenomic analyses, including core-genome-based reconstruction, thereby providing more robust resolution of closely related bacterial taxa [19,20].

Functional genome annotation and targeted analysis of genes associated with nutrient metabolism, carbohydrate degradation, transport systems, and secondary metabolite biosynthesis enable the assessment of the potential ecological and biotechnological functions of bacterial strains [21,22,23]. However, genomic data primarily reflect genetic potential, whereas the functional activity of the identified pathways requires further experimental validation.

Despite the rapidly increasing number of available *Bacillus* genomes, environmental isolates of potential interest for organic substrate bioconversion remain insufficiently characterized with respect to the combined presence of functional systems associated with nitrogen transformation, plant polysaccharide degradation, phosphorus metabolism, and secondary metabolite biosynthesis. Comparative analysis of strains with potentially complementary functional genomic profiles is therefore of particular interest, as the efficient processing of complex organic substrates depends not on a single functional trait but on the combined processes of carbon compound degradation, nutrient transformation, and adaptation to changing substrate conditions.

The present study aimed to comprehensively characterize the genomes of two environmental bacterial strains, RKM-1 and RKM-2, establish their taxonomic positions, and comparatively assess their functional potential related to nutrient and organic matter transformation. Particular attention was given to genetic systems associated with nitrogen and phosphorus metabolism, carbohydrate utilization, glycogen biosynthesis, and secondary metabolite production. The comparative analysis further aimed to identify potentially complementary functional genomic features of the two strains and provide a genomic basis for their subsequent experimental evaluation in organic waste bioconversion and composting.

## 2. Materials and Methods

### 2.1. Isolation and Screening of Bacterial Strains

Thirty-six thermophilic bacterial isolates were isolated from sewage sludge samples at wastewater treatment facilities in the Mangystau region, Kazakhstan. Thermophilic bacteria were isolated by cultivating serial dilutions of the samples on nutrient agar (HiMedia Laboratories Pvt. Ltd., Mumbai, India) at 50 °C [24].

The isolates were evaluated for amylolytic, cellulolytic, chitinolytic and proteolytic, activities using substrate-specific agar media. Amylolytic activity was determined on starch agar by flooding the plates with iodine solution after incubation. Cellulolytic activity was assessed on agar medium containing carboxymethyl cellulose (Sigma-Aldrich, St. Louis, MI, USA), followed by Congo red staining (Sigma-Aldrich, USA) [25]. Chitinolytic activity was evaluated on agar supplemented with colloidal chitin (Sigma-Aldrich Química, S.L., Sintra, Portugal), whereas proteolytic activity was determined on skim milk agar (HiMedia Laboratories Pvt. Ltd., Mumbai, India) [26].

Enzymatic activity was evaluated by measuring the diameter of the hydrolysis zones surrounding bacterial colonies. The hydrolysis index (HI) was calculated as

(1) $$\mathrm{HA}=\frac{\mathrm{Dh}}{\mathrm{Dc}},$$

where Dh is the total diameter of the hydrolysis zone including the colony, and D_C_ is the colony diameter [26].

Based on the overall hydrolytic activity profiles, two bacterial isolates exhibiting the highest biodegradation potential were selected for subsequent pilot-scale composting experiments and whole-genome sequencing. The selected strains were designated *B. subtilis* RKM1 and *B. licheniformis* RKM2.

### 2.2. Preparation of Bacterial Inocula

The selected strains were cultivated separately in liquid nutrient medium ((HiMedia Laboratories Pvt. Ltd., Mumbai, India)) under aerobic conditions at 50 °C for 24 h. Bacterial suspensions were adjusted to approximately 1 × 10^8^ CFU mL^−1^ before application.

Immediately before inoculation, the cultures were diluted with non-chlorinated water to obtain the working suspension required for homogeneous application throughout the composting material.

### 2.3. Composting Experiment

Composting was carried out under pilot-scale conditions at an ambient temperature of approximately 25–30 °C. The composting experiment consisted of three treatments: (i) sewage sludge inoculated with *B. subtilis* RKM-1, (ii) sewage sludge inoculated with *B. licheniformis* RKM-2, and (iii) non-inoculated sewage sludge used as the control.

Each composting pile consisted of 300 kg of sewage sludge mixed with 30% (*w*/*w*) air-dried plant residues used as a structural bulking agent. The components were thoroughly homogenized before pile formation to improve aeration and create favorable conditions for microbial activity.

The bacterial inoculum was evenly sprayed onto the composting material during pile formation, followed by additional mixing to ensure uniform distribution of bacterial cells throughout the pile. The control treatment received the same volume of water without bacterial inoculation.

Composting was performed under natural environmental conditions.

Throughout composting, the piles were periodically turned to improve oxygen penetration, redistribute moisture, homogenize the composting material, and prevent anaerobic conditions. Moisture content was maintained by periodic watering whenever drying of the composting material was observed.

The internal temperature and moisture content of each compost pile were monitored throughout the composting process using a portable digital soil temperature and moisture meter. The probe was inserted into the central part of each pile at a depth of approximately 30–50 cm. Measurements were performed at regular intervals throughout the composting period. The composting experiment was conducted in triplicate, with three independent replicate piles established for each treatment (*n* = 3).

### 2.4. Determination of Total Nitrogen and Organic Carbon

Representative compost samples were collected periodically throughout the composting process. Prior to analysis, the samples were air-dried, homogenized, and prepared for analysis.

Total nitrogen (TN) was determined using the Kjeldahl method [27], whereas organic carbon (OC) was determined by the dichromate oxidation method [28].

The carbon-to-nitrogen ratio (C:N) was calculated from the measured values of organic carbon and total nitrogen using the following equation:

(2) $$\text{C:N}=\frac{\mathrm{TN}}{\mathrm{OC}},$$

where OC is the organic carbon content and TN is the total nitrogen content, both expressed on a dry matter basis.

### 2.5. Whole-Genome Sequencing and Comparative Genomic Analysis

**Figure 1:**
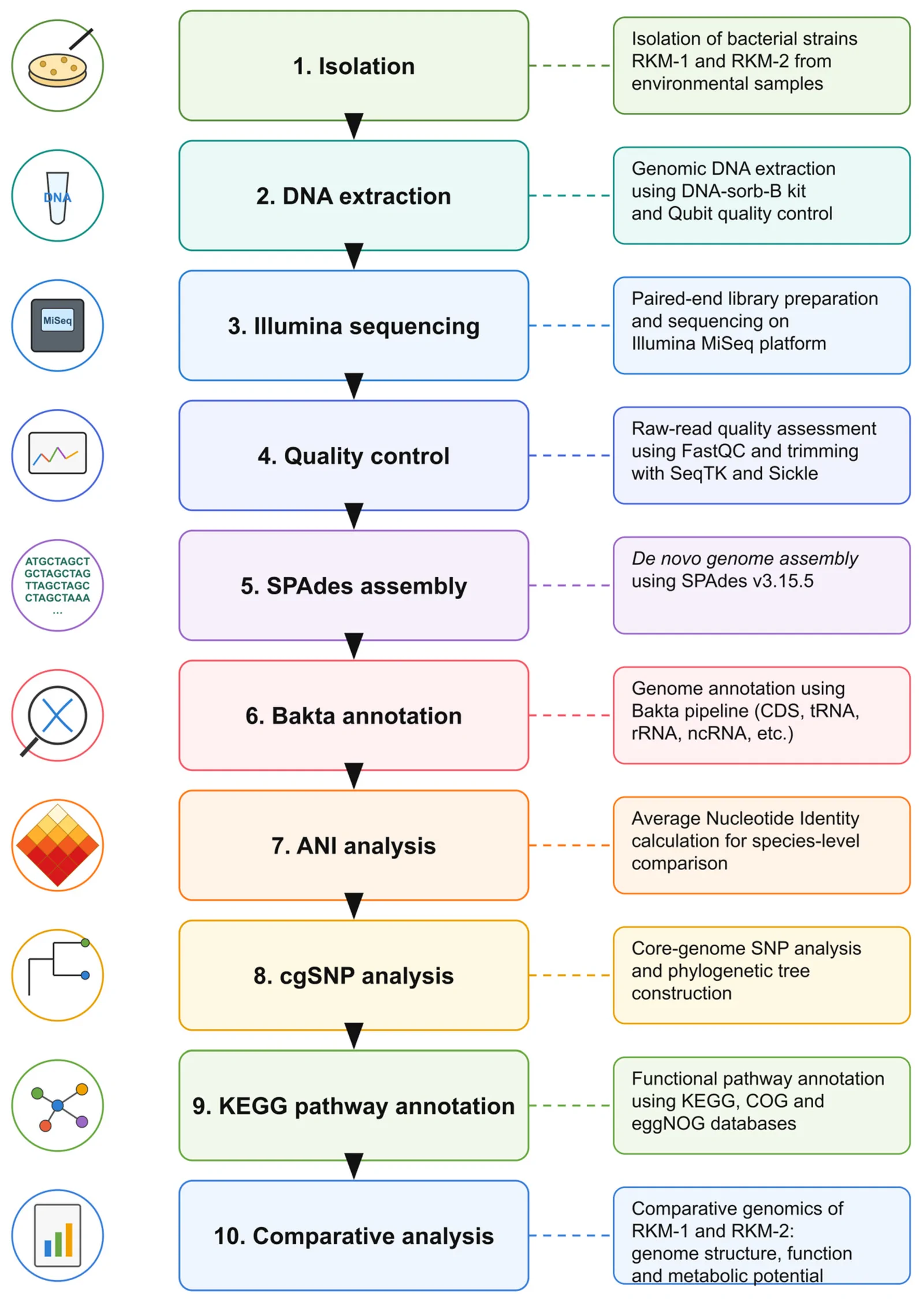
Schematic workflow of the study, including bacterial isolation, whole-genome sequencing, genome assembly, genome annotation, genome-based taxonomic identification (ANI and TYGS), phylogenetic analysis, functional annotation (KEGG, COG, and eggNOG), and comparative genomic analysis.

Among the obtained isolates, RKM-1 and RKM-2 exhibited stable growth under thermophilic cultivation conditions and were subsequently subjected to whole-genome sequencing and comparative genomic analysis. Whole-genome sequencing and comparative genomic analysis of strains RKM 1 and RKM-2 were performed according to the workflow presented in Figure 1. The analytical workflow included bacterial isolation, genomic DNA extraction, Illumina sequencing, sequencing read quality assessment, de novo genome assembly, structural and functional genome annotation, genome-based taxonomic identification, phylogenetic reconstruction and comparative metabolic analyses.

### 2.6. Genomic DNA Extraction

Genomic DNA was extracted from overnight bacterial cultures using the DNA-sorb-B DNA Extraction Kit (AmpliSens, Moscow, Russia). DNA concentration was measured using a Qubit^TM^ 4 fluorometer (Thermo Fisher Scientific, Waltham, MA, USA) with the Qubit^TM^ dsDNA High Sensitivity Assay Kit. DNA purity and integrity were assessed prior to library preparation. Only DNA samples meeting the quality requirements for next-generation sequencing were selected for subsequent analyses.

### 2.7. Whole-Genome Sequencing

Whole-genome sequencing libraries were prepared using the Collibri^TM^ ES DNA Library Prep Kit for Illumina systems (Invitrogen, Waltham, MA, USA). Paired-end sequencing was performed on the Illumina MiSeq platform using the MiSeq Reagent Kit v3 (600-cycle) (Illumina, San Diego, CA, USA) according to the manufacturer’s protocol. Sequencing quality was monitored throughout the sequencing run using standard illumina quality metrics, including cluster density, percentage of clusters passing filter, read quality distribution, and the proportion of bases with a Phred quality score of ≥Q30, ensuring the generation of high-quality sequencing data suitable for downstream genome assembly [17].

### 2.8. Quality Control of Sequencing Reads

Raw sequencing reads were subjected to quality assessment using FastQC v0.12.1 to evaluate per-base sequence quality, GC content, sequence duplication levels, adapter contamination, and overrepresented sequences. Adapter sequences and low-quality bases were removed using SeqTK v.1.4, followed by quality trimming with Sickle v1.33 employing a sliding-window algorithm. Only high-quality paired-end reads that passed all filtering criteria were retained for genome assembly.

### 2.9. Genome Assembly

Filtered sequencing reads were assembled de novo using SPAdes v3.15.5 with default parameters optimized for bacterial genome assembly. Assembly quality was assessed using QUAST v5.2 by evaluating total assembly length, number of contigs, N50 value, GC content, and genome completeness metrics.

### 2.10. Genome Annotation

Structural and functional genome annotation was performed using Bakta v1.9, which predicts protein-coding sequences (CDSs), transfer RNAs (tRNAs), ribosomal RNAs, non-coding RNAs (ncRNAs), CRISPR loci and pseudogenes using curated reference databases. The resulting annotation files were used for all subsequent comparative genomic analyses.

### 2.11. Genome-Based Taxonomic Identification

Genome-based taxonomic identification of strains RKM-1 and RKM-2 was performed using the Type Genome Server (TYGS), which provides automated phylogenomic classification based on whole-genome comparisons. Closely related type strains were identified using the MASH algorithm together with 16S rRNA gene sequence comparisons implemented within the TYGS workflow [20,29].

Phylogenetic distances between genomes were calculated using the Genome BLAST Distance Phylogeny (GBDP) approach [30]. Digital DNA-DNA hybridization (dDDH) values and their confidence intervals were estimated using the Genome-to-Genome Distance Calculator (GGDC 4.0) implemented within TYGS “https://tygs.dsmz.de/ (accessed on 9 June 2026)” [31]. To further confirm species assignment, Average Nucleotide Identity (ANI) values were calculated between the assembled genomes and representative reference genomes of the genus *Bacillus*. ANI values above 95–96% were considered indicative of species-level identity according to current recommendations for prokaryotic taxonomy [32].

### 2.12. Core Genome Phylogenetic Analysis

Comparative phylogenetic analysis was performed using orthologous genes shared among the analyzed genomes. Gene clustering and core genome identification were carried out using Panaroo, which minimizes annotation inconsistencies and improves ortholog prediction [33]. Multiple sequence alignment of concatenated core genes was used for phylogenetic reconstruction with IQ-TREE 2 using the maximum likelihood method [34]. The optimal nucleotide substitution model was selected automatically using ModelFinder [35], whereas branch support was assessed using 1000 ultrafast bootstrap replicates [36] and the SHaLRT test [37].

### 2.13. Functional Genome Annotation

Protein sequences predicted by Bakta were functionally classified using the Kyoto Encyclopedia of Genes and Genomes (KEGG) database to reconstruct metabolic pathways involved in central metabolism, nitrogen transformation, carbohydrate utilization, and phosphorus metabolism [21]. Additional functional classification was performed using the Clusters of Orthologous Groups (COG) database, allowing predicted proteins to be assigned to major cellular functional categories [38].

Ortholog identification and functional genome annotation were further refined using eggNOG-mapper v2, which integrates orthology inference with Gene Ontology (GO), KEGG Orthology (KO), and COG functional assignments [23].

### 2.14. Identification of Genes Involved in Nitrogen and Carbon Metabolism

For nitrogen metabolism, particular attention was given to genes associated with nitrate uptake and reduction (*narG*, *narH*, *narI*, *narJ*, and *narK*), assimilatory nitrate metabolism (*nasA*, *nasB*, *nasC*, and *nasD*), nitrite reduction (*nirB*, *nirC*, and *nirD*), and related transport systems [16].

Genes associated with carbohydrate metabolism included enzymes involved in starch degradation (*amyA* and *amyE*), cellulose degradation (*celA*), glycoside hydrolysis (*glgA*, *glgB*, *glgC*, and *glgD*), sugar interconversion (*galE* and *galU*), and phosphotransferase system-mediated carbohydrate utilization (*glcT*) [39]. Genes associated with phosphate metabolism (phoA) [40] and secondary metabolite biosynthesis (pksA, *pksM*, and *pksR*) [41] were also identified and compared between the two genomes.

### 2.15. Comparative Genomic Analysis and Visualization

Comparative genomic analysis of strains *B. subtilis* RKM1 and *B. licheniformis* RKM2 was performed by integrating genome annotation, phylogenetic reconstruction, and metabolic pathway analysis [36,42]. Circular genome maps illustrating the distribution of coding sequences, GC content, GC skew, and selected functional genes were generated using Proksee [43]. Metabolic pathway diagrams were manually reconstructed in Inkscape v1.4.4 based on annotated genes identified in each genome. Only representative genes involved in nitrogen transformation, carbohydrate utilization, glycogen biosynthesis, phosphate metabolism, and secondary metabolite biosynthesis were included in the final pathway reconstruction. All graphical illustrations were prepared in publication-quality vector format and edited to ensure consistency of fonts, colors, and graphical elements.

## 3. Results

### 3.1. Extracellular Enzyme Activity of Bacterial Isolates

**Figure 2:**
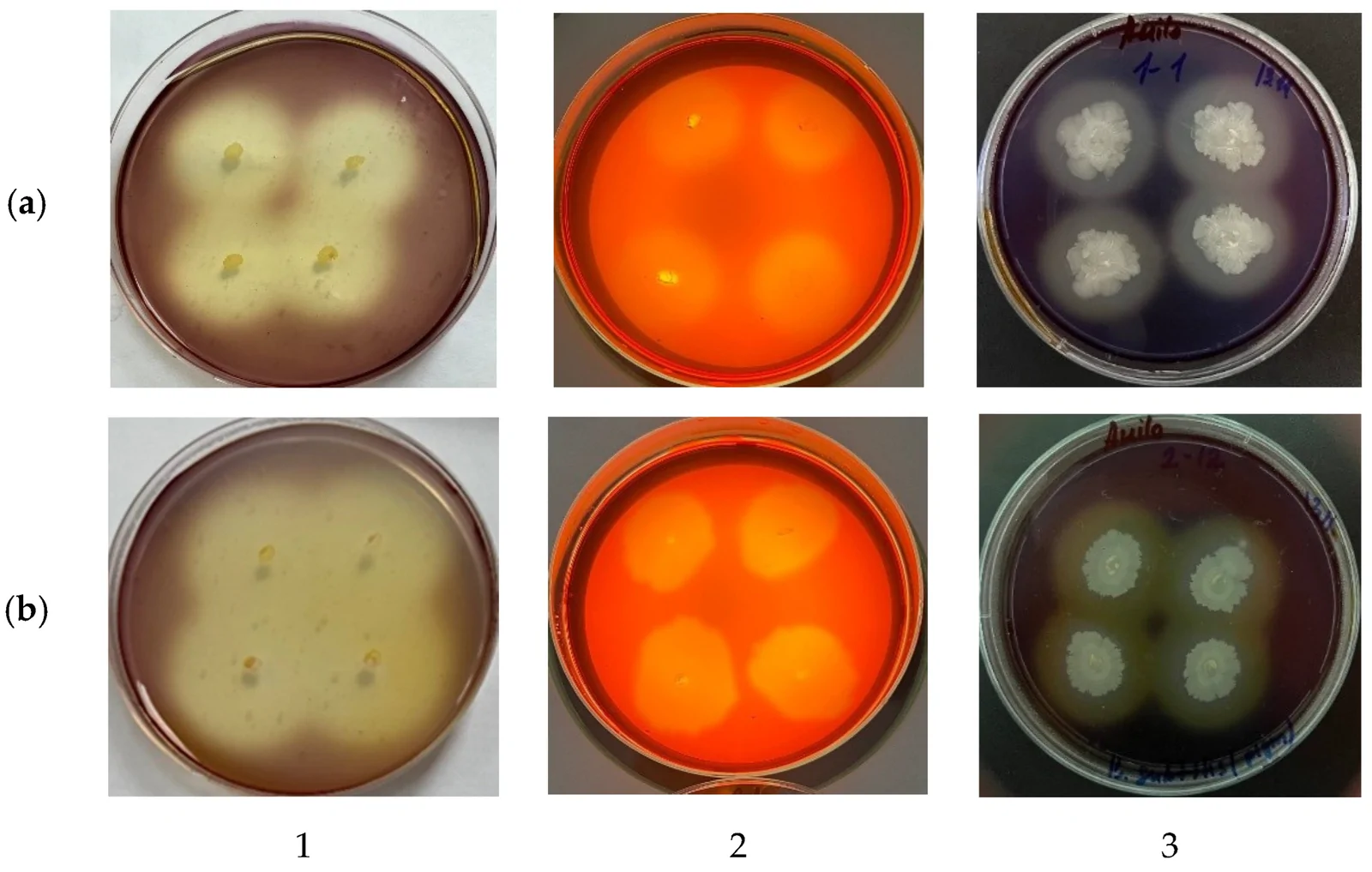
Extracellular enzymatic activities of *B. subtilis* RKM-1 (**a**) and *B. licheniformis* RKM-2 (**b**): 1—chitinolytic; 2—cellulolytic; 3—amylolytic.

Among the 36 thermophilic bacterial isolates screened *B. subtilis* RKM-1 and *B. licheniformis* RKM-2 exhibited the highest extracellular enzymatic activities, including cellulolytic, chitinolytic and amylolytic activities (Table 1) (Figure 2).

Cellulolytic activity was the predominant extracellular enzymatic activity detected in both strains. The cellulase enzymatic index (EI) of *B. licheniformis* RKM-2 reached 8.8 ± 0.29, exceeding that of *B. subtilis* RKM-1 (5.5 ± 0.79) indicating a greater capacity of *B. licheniformis* RKM-2 to degrade cellulose-containing substrates. Both isolates also exhibited high chitinolytic activity. The highest chitinase EI was recorded for *B. licheniformis* RKM-2 (8.5 ± 0.00), whereas *B. subtilis* RKM-1 exhibited an EI of 6.9 ± 0.95 demonstrating the strong chitin-degrading potential of both thermophilic strains.

Amylolytic activity was detected in both isolates, with comparable EI values of 2.5 ± 0.21 for *B. subtilis* RKM-1 and 2.2 ± 0.03 for *B. licheniformis* RKM-2, indicating similar starch-degrading capabilities.

### 3.2. Composting Performance of Thermophilic Bacillus Strains

The composting performance of the selected strains was subsequently evaluated by monitoring temperature dynamics and changes in the C:N ratio during the composting process.

**Figure 3:**
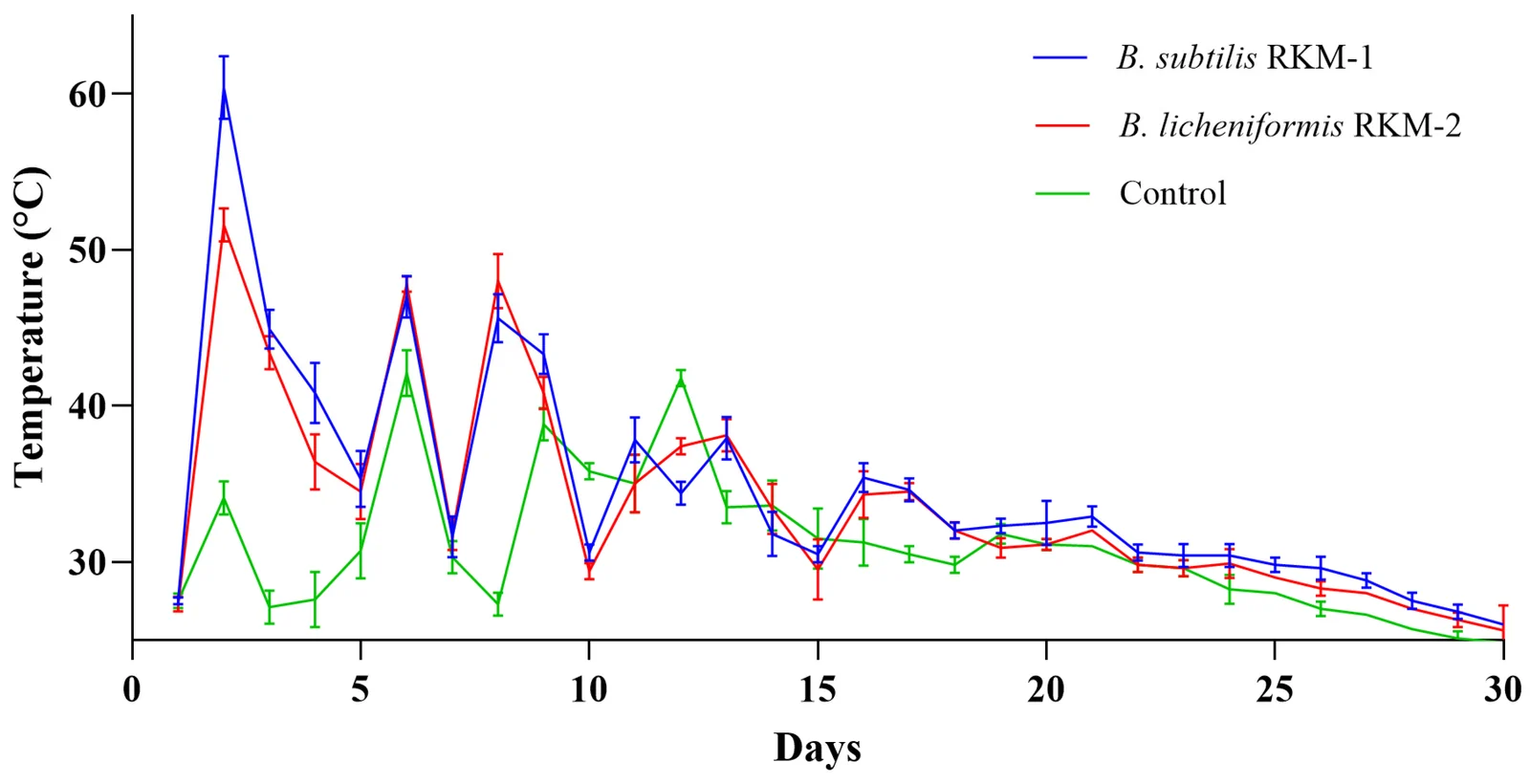
Temperature dynamics during sewage sludge composting with plant residues inoculated with two *Bacillus* strains and the non-inoculated control.

Temperature profiles differed markedly among the treatments (Figure 3). Both inoculated variants rapidly entered the thermophilic phase, whereas the control exhibited a delayed and less pronounced temperature increase. The highest temperature was recorded in the *B. licheniformis* RKM-2 treatment (60 ± 2.00 °C on day 2), followed by *B. subtilis* RKM-1 (51 ± 1.06 °C). In contrast, the control reached a maximum temperature of only 42 ± 1.49 °C and entered the thermophilic phase later, indicating lower microbial activity and slower decomposition of organic matter. During the first two weeks, both inoculated treatments showed several secondary temperature peaks, reflecting sustained microbial metabolism. Thereafter, temperature gradually declined in all treatments, approaching ambient values by the end of the monitoring period, which indicated the transition to the maturation phase.

Temperature profiles differed markedly among the treatments (Figure 3). Both inoculated variants rapidly entered the thermophilic phase, whereas the control exhibited a delayed and less pronounced temperature increase. The highest temperature was recorded in the *B. subtilis* RKM-1 treatment (60 ± 2.00 °C on day 2), followed by *B. licheniformis* RKM-2 (51 ± 1.06 °C). In contrast, the control reached a maximum temperature of only 42 ± 1.49 °C and entered the thermophilic phase later, indicating lower microbial activity and slower decomposition of organic matter. During the first two weeks, both inoculated treatments showed several secondary temperature peaks, reflecting sustained microbial metabolism. Thereafter, temperature gradually declined in all treatments, approaching ambient values by the end of the monitoring period, which indicated the transition to the maturation phase.

**Figure 4:**
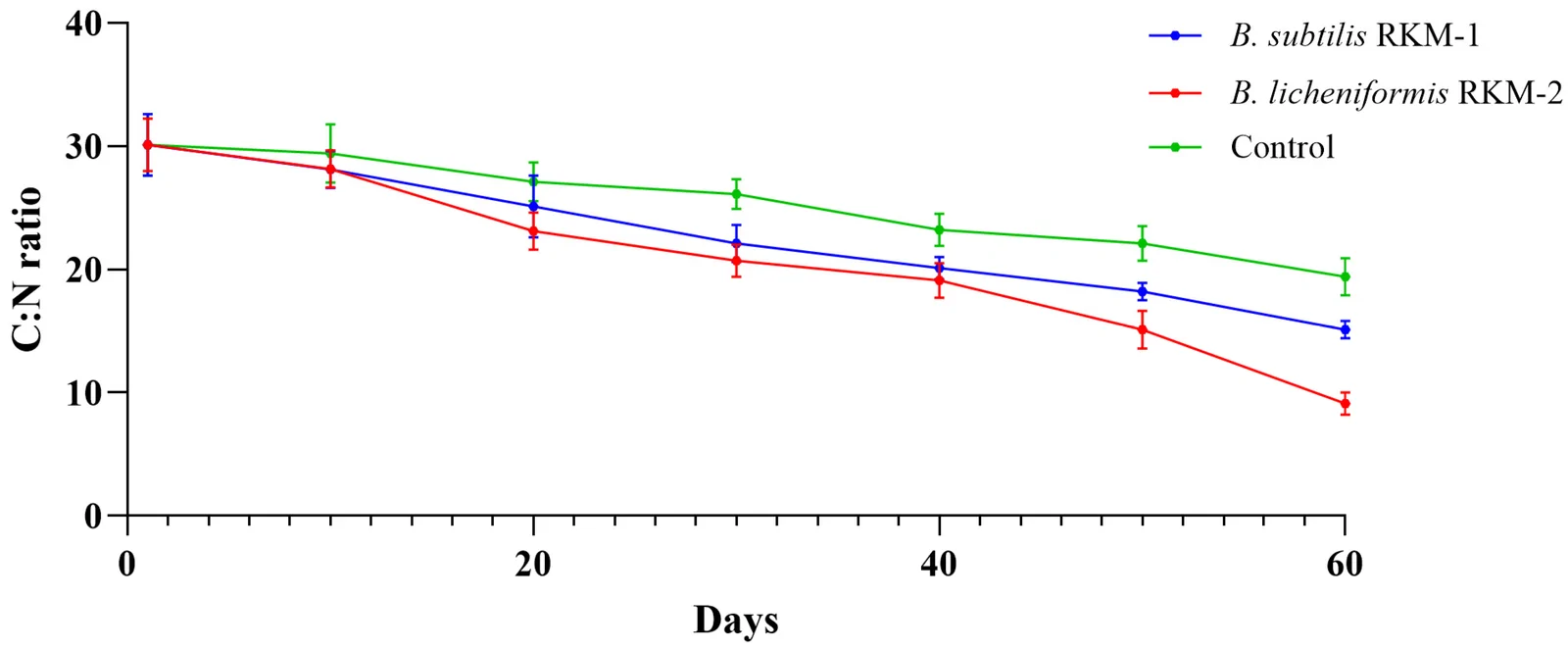
C:N ratio during sewage sludge composting with plant residues inoculated with two Bacillus strains and the non-inoculated control.

The C:N ratio decreased throughout the 60-day composting period in all treatments (Figure 4); however, the rate of reduction differed substantially. The most pronounced decline was observed in the *B. licheniformis* RKM-1 treatment, where the C:N ratio decreased from 9.1 ± 0.91 by day 60. In contrast, the control showed only a gradual decrease with the C:N ratio remaining close to 19.4 ± 1.5 at the end of the experiment. The accelerated reduction in the C:N ratio in the inoculated composts indicates enhanced organic carbon mineralization and faster compost stabilization. Although *B. subtilis* RKM-2 generated the highest thermophilic temperatures, *B. licheniformis* RKM-1 achieved the greatest reduction in the C:N ratio, suggesting distinct yet complementary contributions of the two strains to the composting process.

**Figure 5:**
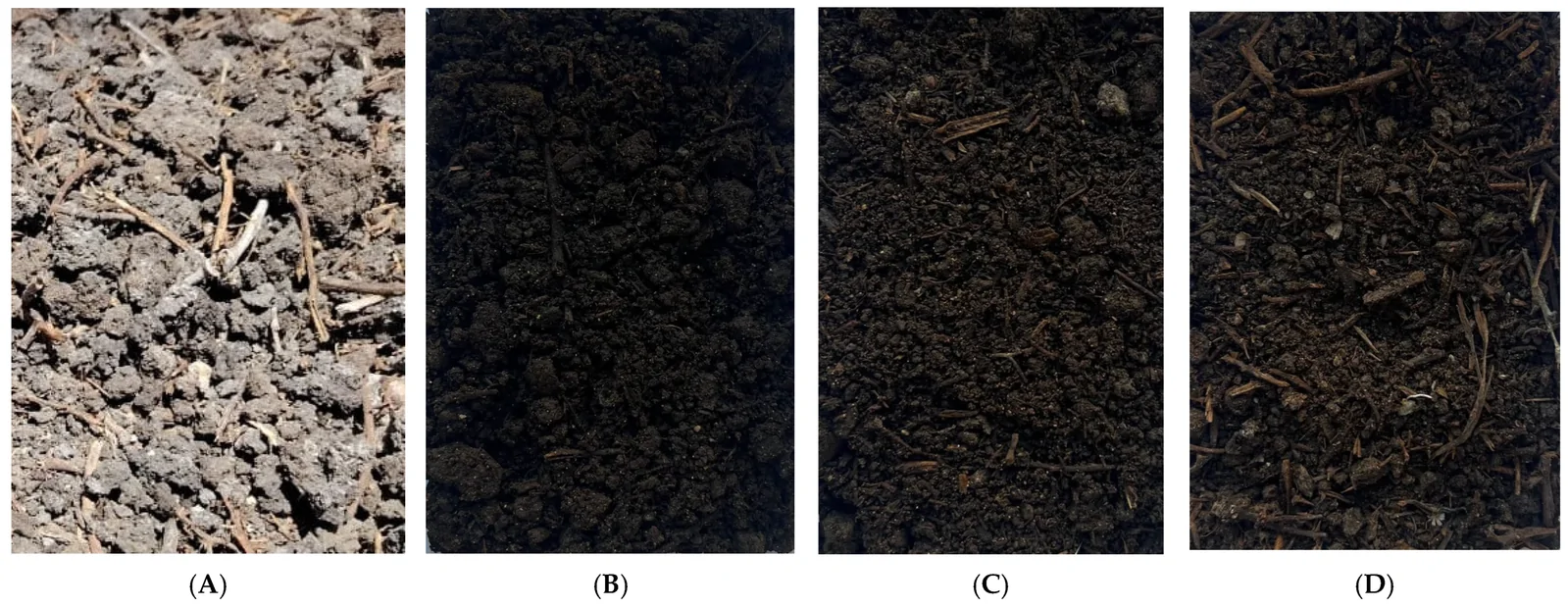
Sewage sludge mixed with dry plant residues: (**A**) before bacterial inoculation; (**B**) after 60 days of composting with B. licheniformis; (**C**) after 60 days of composting with *B. subtilis*; (**D**) after 60 days of composting without bacterial inoculation (control).

Visual examination demonstrated progressive decomposition of the initial substrate in all treatments after 60 days (Figure 5). Compared with the initial mixture, the inoculated composts developed a darker color and a more homogeneous, crumbly structure with fewer visible plant residues. In contrast, the control treatment retained more recognizable fragments of dry plant material and a less uniform texture, consistent with the lower microbial activity indicated by the temperature profiles and the smaller reduction in the C:N ratio.

### 3.3. Whole-Genome Sequencing and Genome Assembly

Whole-genome sequencing of strains RKM-1 and RKM-2 generated high-quality paired-end Illumina reads suitable for de novo genome assembly. After quality filtering and trimming the reads were assembled using the SPAdes assembler, resulting in draft genome assemblies with high sequence continuity and sufficient genome coverage. Assembly quality assessment using QUAST demonstrated that both genomes exhibited characteristics expected for members of the genus *Bacillus*, including an appropriate genome size, GC content, and assembly completeness. Both genome assemblies contained a complete set of protein-coding genes, together with transfer RNA (tRNA), ribosomal RNA (rRNA), and other structural RNA genes predicted using the Bakta annotation pipeline.

The general assembly and annotation characteristics of the strains RKM-1 and RKM-2 are summarized in Table 2.

The genome of strain *B. subtilis* RKM-1 had a total size of 4.06 Mb with a GC content of 43.7% and was assembled into 16 contigs, exhibiting a high N50 value of 2.09 Mb, indicative of high assembly continuity. In contrast, *B. licheniformis* strain RKM-2 possessed a slightly larger genome (4.21 Mb) and a higher GC content (46.1%), but showed lower continuity, with an N50 value of 769 kb. Both genomes contained more than 4000 coding DNA sequences (CDSs), and no plasmids were detected, suggesting a stable genomic architecture.

### 3.4. Genome Annotation

Genome annotation identified several thousand protein-coding sequences in each strain, together with structural RNA genes and other functional genomic elements. Functional classification using the KEGG, COG, and eggNOG databases assigned the majority of the predicted proteins to conserved metabolic pathways involved in central cellular metabolism, genetic information processing, and environmental adaptation.

**Figure 6:**
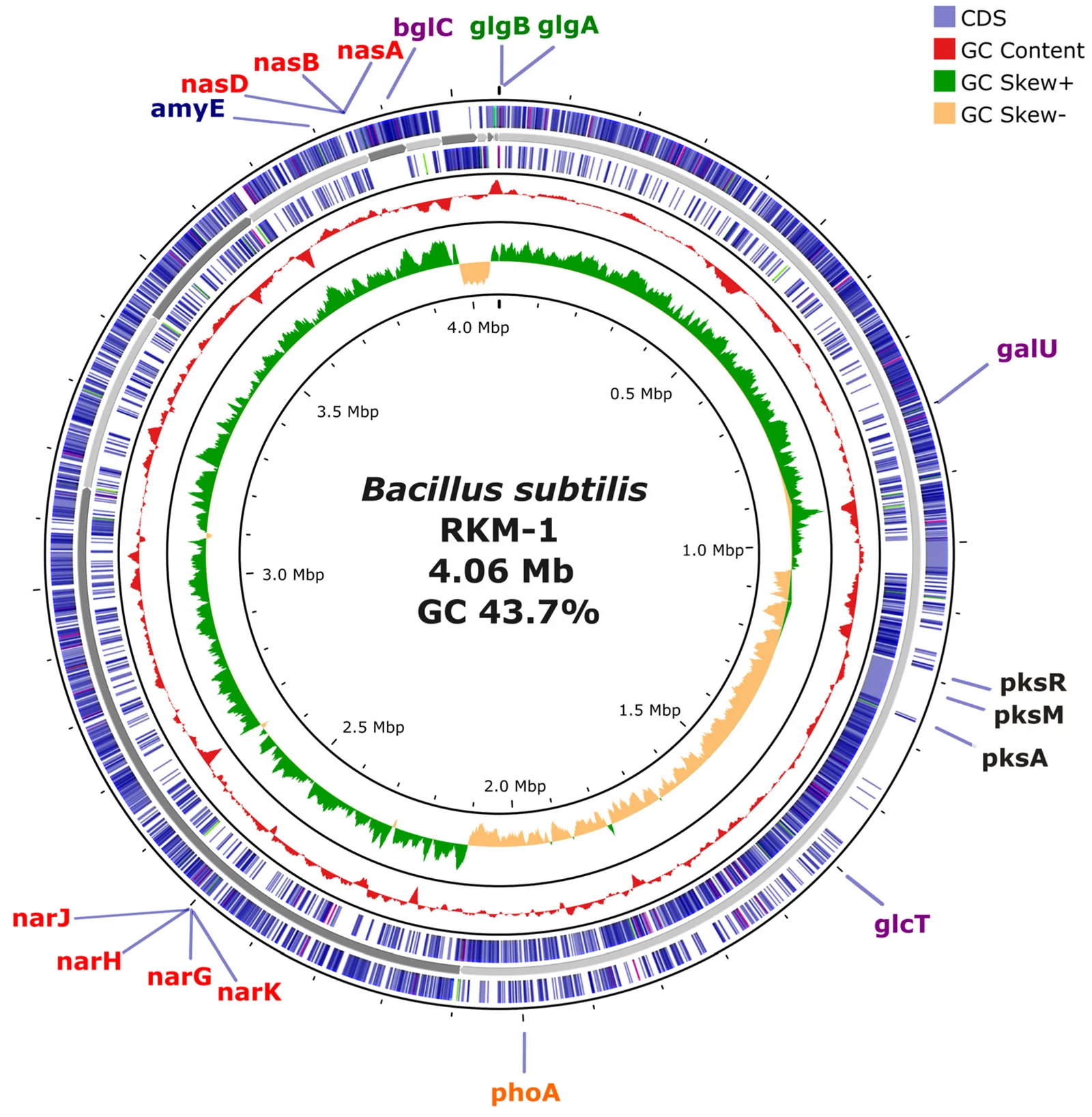
Circular genome map of *B. subtilis* strain RKM-1 generated using Proksee. The chromosome is 4.06 Mb in length with an average GC content of 43.7%. From the outermost to the innermost rings: predicted coding DNA sequences (CDSs) on the forward strand, CDSs on the reverse strand, GC content, and GC skew. Representative genes involved in nitrogen metabolism (*narG*, *narH*, *narJ*, *narK*, *nasA*, *nasB*, and *nasD*), carbohydrate metabolism (*amyE* and *bglC*), glycogen biosynthesis (*glgA* and *glgB*), phosphate metabolism (*phoA*), UDP-sugar metabolism (*galU*), and polyketide biosynthesis (*pksA*, *pksM*, and *pksR*) are indicated.

**Figure 7:**
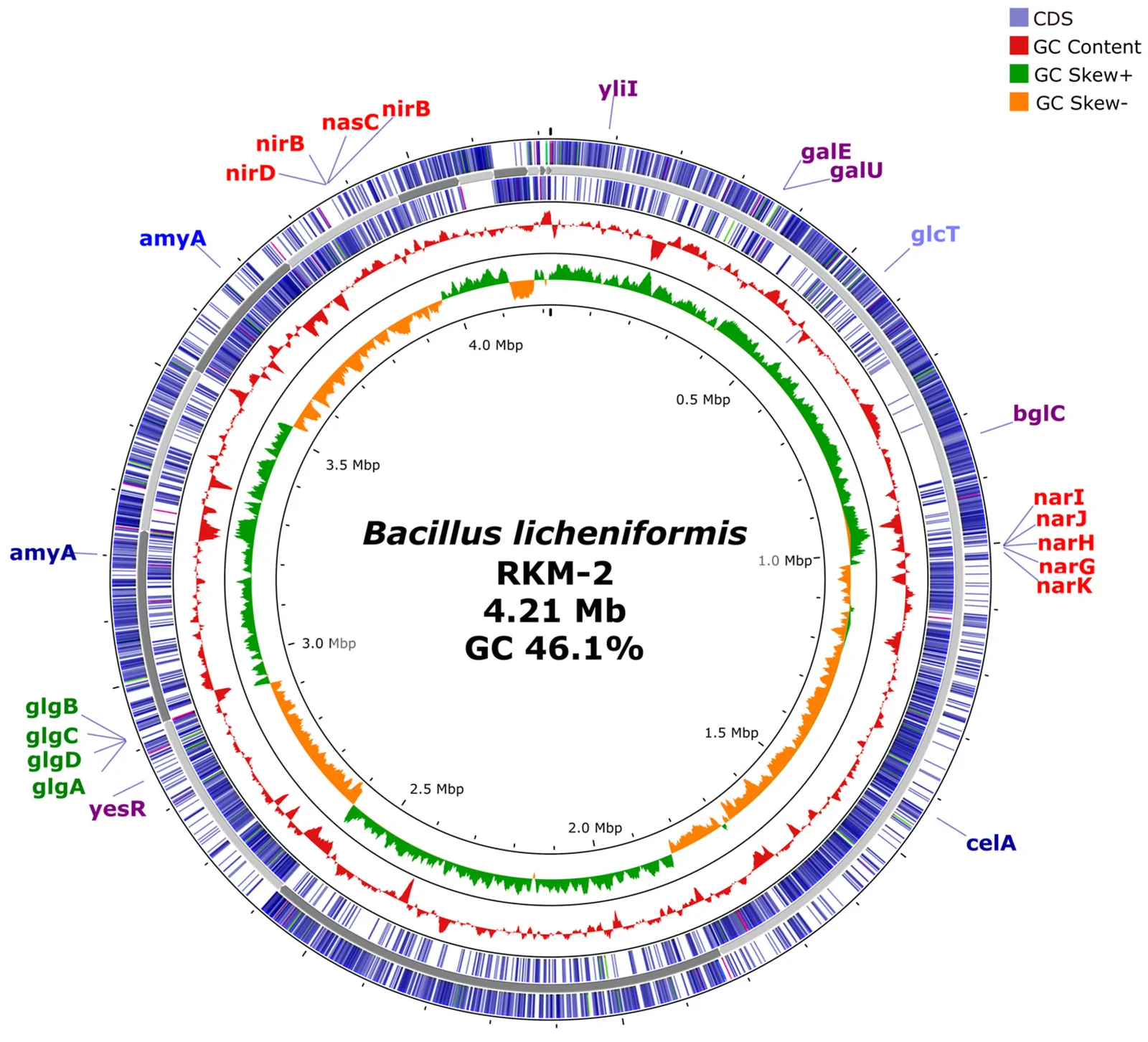
Circular genome map of *B. licheniformis* strain RKM-2 generated using Proksee. The chromosome has a total length of 4.21 Mb with an average GC content of 46.1%. From the outermost to the innermost rings: predicted coding DNA sequences (CDSs) on the forward strand, CDSs on the reverse strand, GC content, and GC skew. Representative genes associated with the major metabolic pathways identified in the genome are highlighted, including genes involved in nitrogen metabolism (*narG*, *narH*, *narI*, *narJ*, *narK*, *nasC*, *nirB*, and *nirD*), carbohydrate utilization (*amyA*, *bglC*, *celA*, and *glcT*), glycogen biosynthesis (*glgA*, *glgB*, *glgC*, and *glgD*), UDP-sugar metabolism (*galE* and *galU*), and glycoside hydrolysis (*yesR*).

Genes involved in amino acid metabolism, carbohydrate metabolism, membrane transport, energy production, and transcription represented the largest functional categories in both genomes. In addition, numerous genes associated with stress adaptation, sporulation, and nutrient utilization were identified, reflecting the ecological versatility characteristic of members of the genus *Bacillus*. Circular genome maps illustrating chromosome organization, GC content, GC skew, and the distribution of representative functional genes are presented in Figure 6 and Figure 7.

### 3.5. Genome-Based Taxonomic Identification

**Figure 8:**
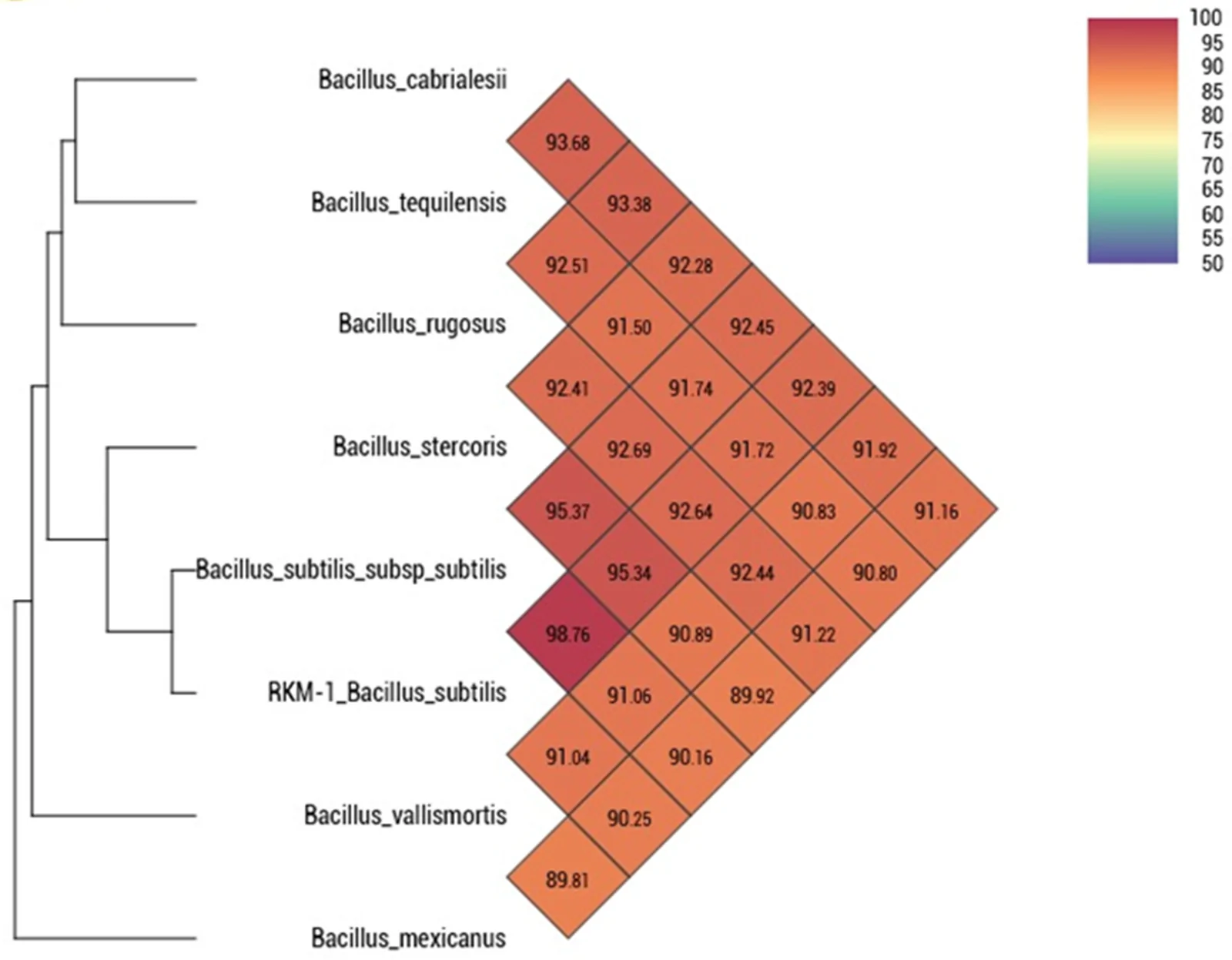
Comparative genome-based analysis of strain RKM-1 using ANI and dDDH relative to closely related *Bacillus* species.

**Figure 9:**
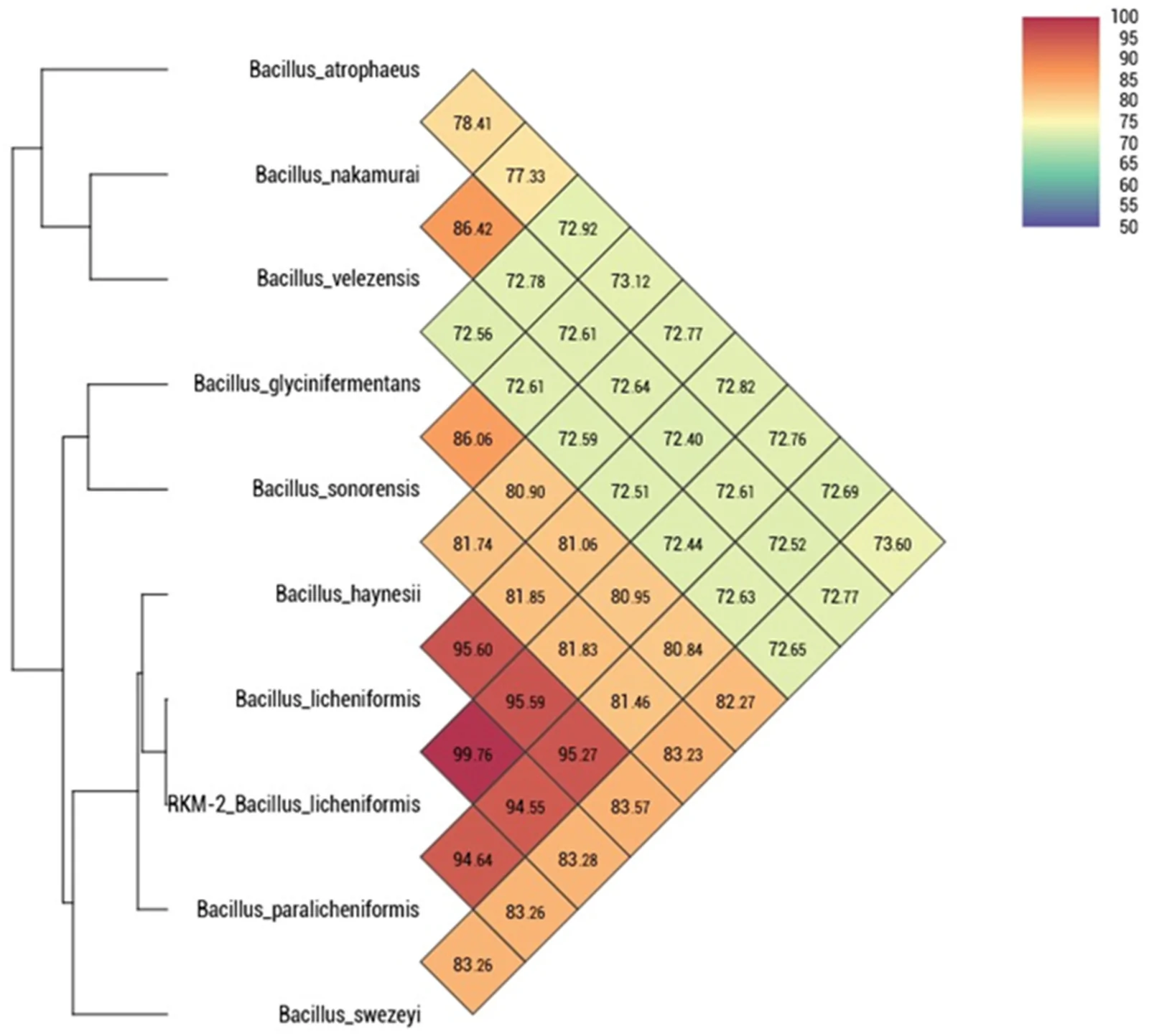
Comparative genome-based analysis of strain RKM-2 using ANI and dDDH relative to closely related *Bacillus* species.

To determine the taxonomic identity of the studied isolates, genome-based species identification was performed using Average Nucleotide Identity (ANI) analysis and the Type (Strain) Genome Server (TYGS) phylogenetic framework. Strain RKM-1 exhibited the highest ANI value (98.76%) relative to reference genomes of *B. subtilis*, clearly exceeding the accepted species threshold of 95–96% (Figure 8). Similarly, strain RKM-2 showed an ANI value of 99.76% compared with reference genomes of *B. licheniformis* (Figure 9), confirming its assignment to this species.

Genome-based taxonomic analysis using TYGS produced consistent results. Digital DNA–DNA hybridization (dDDH) values exceeded the accepted species delineation threshold, while phylogenetic clustering placed both isolates within the corresponding clades of their respective type strains, providing independent confirmation of the ANI-based classification [21,31].

These results demonstrate that strains RKM-1 and RKM-2 belong to the species *B. subtilis* and *B. licheniformis*, respectively.

### 3.6. Comparative Phylogenomic Analysis

**Figure 10:**
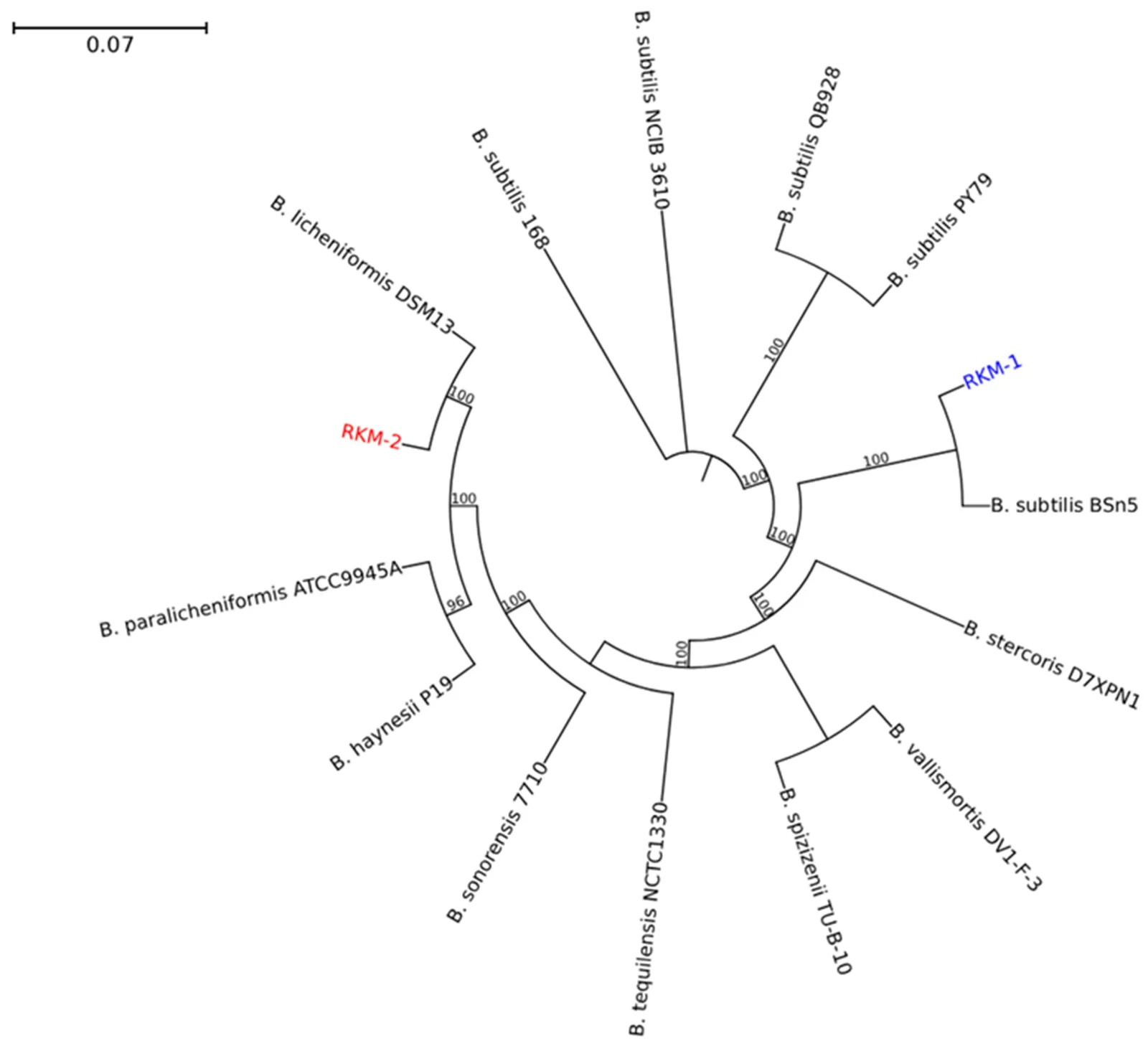
Maximum-likelihood phylogenomic tree constructed from the concatenated core genome alignment of representative *Bacillus* species. Phylogenomic reconstruction was performed using IQ-TREE 2 with the best-fit evolutionary model selected by ModelFinder. Node values indicate Ultrafast Bootstrap support based on 1000 pseudoreplicates. Branch lengths represent the number of nucleotide substitutions per site. The studied strains RKM-1 (blue) and RKM-2 (red) are highlighted.

To further investigate the evolutionary relationships of the studied isolates, a genome-scale phylogenetic analysis was performed using the concatenated core genomes of representative members of the *B. subtilis* species complex and closely related taxa (Figure 10). The maximum-likelihood phylogenetic tree clearly separated the analyzed genomes into two distinct evolutionary lineages corresponding to their taxonomic affiliations.

Strain RKM-1 clustered within the *B. subtilis* lineage and exhibited the closest phylogenetic relationship to *B. subtilis* BSn5, supported by the maximum bootstrap value (100%). Despite this close relationship, strain RKM-1 formed a distinct branch separated from other representative *B. subtilis* strains, including PY79, QB928, NCIB 3610, and strain 168. Strain RKM-2 clustered within the *B. licheniformis* lineage and showed the closest phylogenetic relationship to *B. licheniformis* DSM 13, also supported by a bootstrap value of 100%. The corresponding clade additionally included *B. paralicheniformis* ATCC 9945A and *B. haynesii* P19, whereas *B. sonorensis* occupied a more distantly related evolutionary position.

Most internal branches of the phylogenetic tree were supported by bootstrap values ranging from 89% to 100%, indicating a robust and well-resolved phylogenetic topology. The reconstructed phylogeny was fully consistent with the ANI and digital DNA–DNA hybridization (dDDH) analyses, providing independent genome-scale evidence supporting the taxonomic classification of both isolates.

Overall, the phylogenomic reconstruction confirmed that strain RKM-1 belongs to the *B. subtilis* lineage, whereas strain RKM-2 belongs to *B. licheniformis*, and accurately resolved their evolutionary relationships with closely related members of the genus.

### 3.7. Functional Genome Annotation

Functional annotation using the KEGG, COG, and eggNOG databases revealed a broad metabolic repertoire in both analysed genomes. Most predicted proteins were assigned to pathways associated with cellular metabolism, genetic information processing, transport systems, and environmental adaptation. Functional classification showed that genes involved in amino acid metabolism, carbohydrate metabolism, energy production, and transport and catabolism represented the major functional categories in both strains. These pathways constitute the core metabolic framework required for bacterial growth and adaptation to diverse environmental conditions.

Despite the overall similarity in global functional composition, several differences were observed between the two genomes. *B. subtilis* RKM-1 contained genes associated with secondary metabolite biosynthesis, including the polyketide synthase gene cluster (*pksA*, *pksM*, and *pksR*), whereas this biosynthetic system was absent from the genome of *B. licheniformis* RKM-2.

In contrast, strain *B. licheniformis* RKM-2 possessed a broader repertoire of enzymes involved in carbohydrate metabolism, including genes associated with cellulose degradation and the utilization of complex carbohydrates. These findings indicate functional specialization of the two isolates despite their close evolutionary relationship within the genus *Bacillus*.

**Figure 11:**
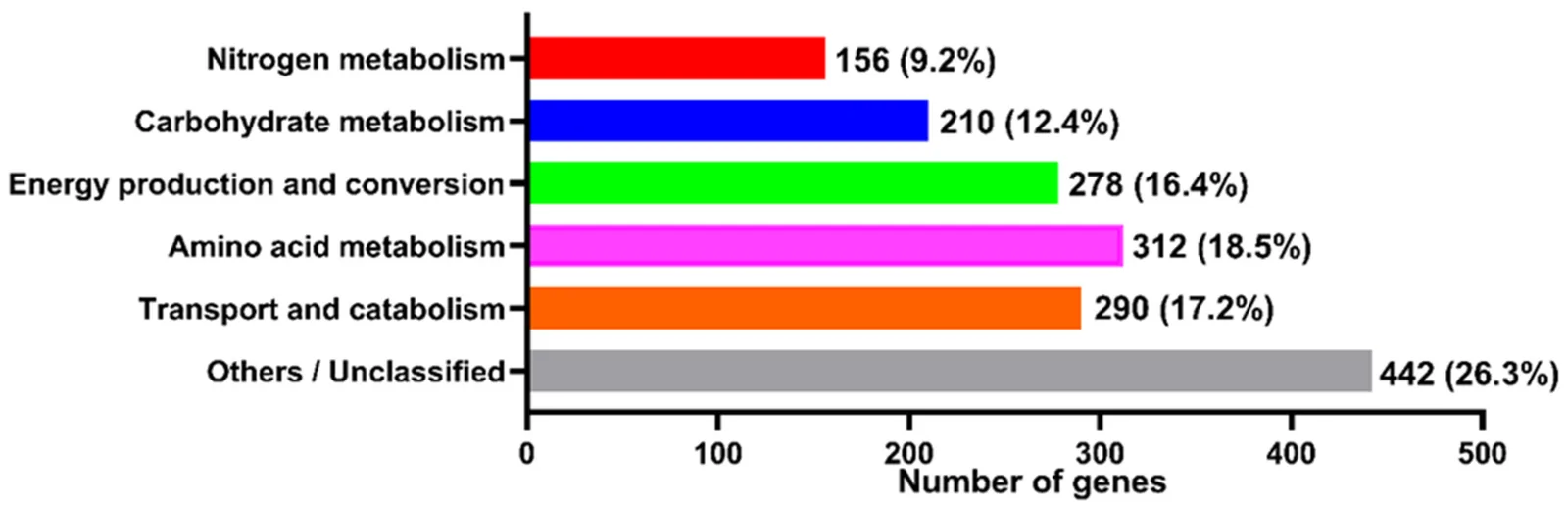
Functional annotation of the *B. subtilis* RKM-1 genome based on the major KEGG functional categories. The distribution of predicted protein-coding genes among the principal KEGG functional categories is shown as the absolute number of genes and their relative proportion (%) of the total annotated genes.

**Figure 12:**
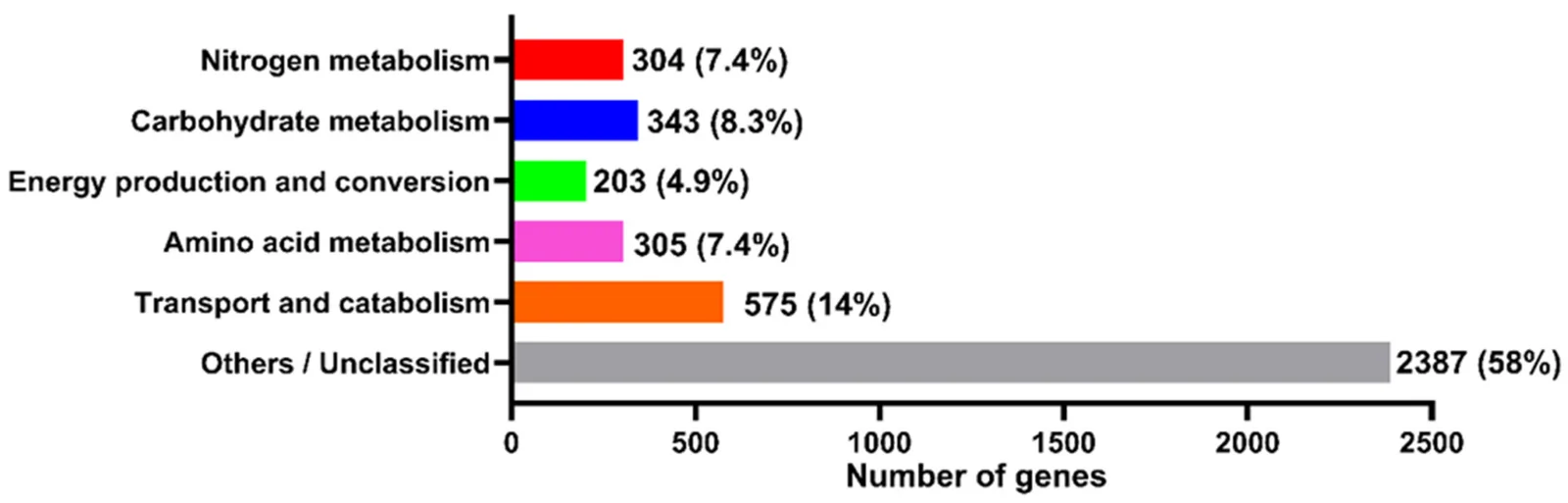
Functional annotation of the *B. licheniformis* RKM-2 genome based on the major KEGG functional categories. The distribution of predicted protein-coding genes among the principal KEGG functional categories is shown as the absolute number of genes and their relative proportion (%) of the total annotated genes.

The distribution of functional categories identified through genome annotation is presented in Figure 11 and Figure 12.

### 3.8. Comparative Analysis of Nitrogen Metabolism

Comparative genomic analysis revealed substantial differences in the organization of nitrogen metabolism between the two strains (Figure 2, Figure 3 and Figure 11).

In *B. subtilis* RKM-1, the nitrate reduction system consisted of the membrane-associated nitrate reductase genes *narG*, *narH*, *narJ*, and *narK*, together with the assimilatory nitrate reduction genes *nasA*, *nasB*, and *nasD*. This genetic organization suggests that nitrate can be transported into the cell, reduced to nitrite, and subsequently assimilated into reduced nitrogen-containing compounds required for cellular biosynthesis.

In contrast, *B. licheniformis* RKM-2 possessed the narGHIJK nitrate reductase operon together with the assimilatory nitrate reductase gene *nasC* and the nitrite reductase genes *nirB*, *nirD*, and *nirC*. The presence of these additional nitrite reduction genes suggests an enhanced capacity for the intracellular conversion of nitrite into ammonium, thereby expanding the potential pathways for nitrogen assimilation.

Although both genomes encode nitrate reduction systems, their genetic organization differs considerably, indicating species-specific strategies for nitrogen utilization.

### 3.9. Comparative Genomic Analysis of Carbon Metabolism

Genes involved in carbohydrate utilization were identified in both genomes; however, their composition differed substantially (Figure 6, Figure 7 and Figure 9). The genome of *B. subtilis* RKM-1 contained the α-amylase gene *amyE*, the β-glucosidase gene *bglC*, the glycogen biosynthesis genes *glgA* and *glgB*, the phosphate metabolism gene *phoA*, and the *pksA*–*pksM*–*pksR* gene cluster responsible for polyketide biosynthesis.

In comparison, *B. licheniformis* RKM-2 possessed a considerably broader repertoire of carbohydrate-related genes, including *amyA*, *celA*, *bglC*, *glcT*, *galE*, *galU*, *glgA*, *glgB*, *glgC*, *glgD*, and *yesR*. The presence of *celA* suggests the potential ability to hydrolyze cellulose-derived substrates, whereas *galE* and *galU* are involved in UDP-sugar metabolism and carbohydrate interconversion. The expanded glycogen biosynthesis operon (*glgABCD*) further suggests an enhanced capacity for carbon storage compared with strain RKM-1. These genomic differences indicate that *B. licheniformis* RKM-2 possesses a more versatile carbohydrate utilization system, whereas *B. subtilis* RKM-1 appears to allocate a greater proportion of its metabolic potential to secondary metabolite biosynthesis.

### 3.10. Comparative Reconstruction of Nitrogen and Carbon Metabolic Pathways

**Figure 13:**
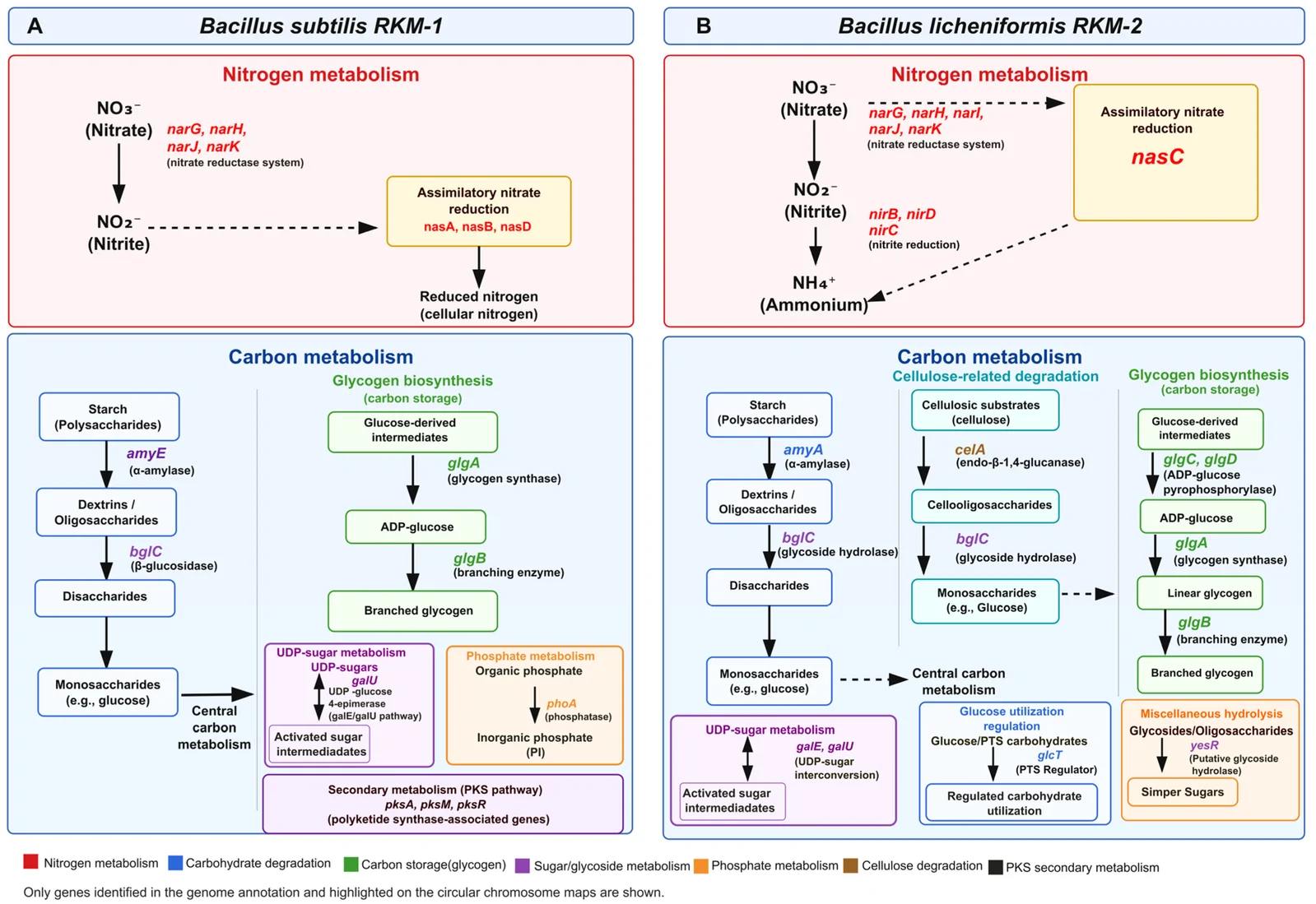
Comparative reconstruction of nitrogen and carbon metabolic pathways encoded by the genomes of *B. subtilis* RKM-1 (**A**) and *B. licheniformis* RKM-2 (**B**). Metabolic pathways were reconstructed based on genome annotation. Only representative genes identified through genome annotation and highlighted on the circular chromosome maps are shown.

To better understand the functional potential of the studied strains, genes involved in nitrogen assimilation, carbohydrate utilization, glycogen biosynthesis, phosphate metabolism, and secondary metabolite production were integrated into a comparative metabolic reconstruction (Figure 13). This analysis revealed both conserved metabolic pathways shared by the two strains and species-specific functional adaptations that may determine their ecological roles and potential biotechnological applications.

Both genomes encoded the membrane-associated nitrate reductase complex (narGHIJK), indicating the ability to utilize nitrate as an alternative nitrogen source under appropriate environmental conditions [13,40,44]. However, the nitrogen assimilation pathways differed between the two strains. In *B. subtilis* RKM-1, nitrate assimilation was primarily represented by the genes *nasA*, *nasB*, and *nasD*, whereas *B. licheniformis* RKM-2 contained the assimilatory nitrate reductase gene nasC together with the nitrite reductase genes *nirB*, *nirC*, and *nirD*, suggesting a broader genetic capacity for intracellular nitrite reduction and ammonium production [40,45].

More pronounced differences were observed in carbon metabolism. Although both strains possessed genes associated with starch utilization and glycogen biosynthesis, *B. licheniformis* RKM-2 contained a substantially expanded repertoire of carbohydrate metabolism enzymes. In addition to the α-amylase gene *amyA* and the β-glucosidase gene *bglC*, its genome encoded the cellulase gene *celA*, the carbohydrate transport regulator *glcT*, the UDP-sugar metabolism genes *galE* and *galU*, and the complete glycogen biosynthesis operon (*glgABCD*). These genes are involved in the degradation of plant-derived polysaccharides, carbohydrate interconversion, and intracellular carbon storage [46,47,48,49].

In contrast, the genome of *B. subtilis* RKM-1 contained the genes *amyE*, *bglC*, *glgA*, *glgB*, and *phoA*, but lacked *celA* and several additional carbohydrate utilization genes identified in RKM-2. Instead, *B. subtilis* RKM-1 possessed the complete *pksA*–*pksM*–*pksR* biosynthetic gene cluster encoding a type I polyketide synthase, indicating the potential capacity to synthesize bioactive secondary metabolites [11,42,50].

The presence of *phoA* in *B. subtilis* RKM-1 also suggests an enhanced potential for the mobilization of organic phosphorus through alkaline phosphatase activity, which may contribute to phosphorus availability under nutrient-limited conditions [14,51].

Overall, the comparative metabolic reconstruction demonstrates complementary ecological strategies of the two strains. *B. subtilis* RKM-1 appears to be genetically adapted for nutrient mobilization and secondary metabolite biosynthesis, whereas *B. licheniformis* RKM-2 possesses a broader enzymatic repertoire for the degradation of complex carbohydrates and the turnover of plant-derived organic matter. These characteristics suggest that the two strains may complement each other during the decomposition of organic substrates and therefore represent promising candidates for microbial formulations intended to enhance vermicompost quality and biofertilizer production.

## 4. Discussion

The results of the enzyme assays and composting experiments performed in the present study demonstrated pronounced functional differences between the two investigated strains. *B. licheniformis* RKM-2 exhibited the highest cellulolytic activity and produced the greatest reduction in the C:N ratio during composting, whereas *B. subtilis* RKM-1 reached the highest thermophilic temperature. The more rapid transition of the inoculated treatments to the thermophilic phase compared with the uninoculated control indicates early activation of microbial metabolism following strain inoculation. The fluctuations observed in the temperature profiles during the active composting stage can be explained by the dynamic nature of microbial heat production and by changes in aeration, moisture distribution, and substrate availability. Periodic turning of the compost piles redistributes oxygen, moisture, and composting material and may temporarily alter heat accumulation, whereas renewed microbial activity following aeration may subsequently result in secondary temperature increases [50]. Spatial temperature gradients may also develop within compost piles because heat generation is generally greater in metabolically active internal zones, while peripheral regions are more strongly influenced by heat exchange with the surrounding environment [51]. In the present study, temperature was measured in the central part of each pile at a depth of approximately 30–50 cm; therefore, the recorded values primarily represent the thermal conditions of the active core. Spatial temperature gradients within individual piles were not specifically quantified and therefore cannot be excluded.

Moreover, the greater reduction in the C:N ratio observed in the *B. licheniformis* RKM-2 treatment suggests more efficient mineralization of organic carbon and accelerated compost maturation. Similar relationships between enhanced microbial activity, organic carbon degradation, and accelerated compost maturation have been reported in previous composting studies [12,52].

The observed functional differences were well supported by the results of comparative whole-genome analysis. The integration of whole-genome sequencing with comparative genomics has become the current standard for investigating the taxonomy, evolutionary relationships, and functional potential of bacterial isolates [20,31,32]. The high level of agreement among ANI, digital DNA-DNA hybridization (dDDH), and phylogenomic analyses confirmed the taxonomic assignment of the investigated isolates to *B. subtilis* and *B. licheniformis* and further demonstrated the reliability of genome-based classification compared with conventional phenotypic approaches [19]. Both strains clustered with their corresponding type and reference genomes while occupying distinct phylogenetic positions within their respective lineages. Similar intraspecific genomic variation has previously been reported for environmental isolates of *B. subtilis* and B. *licheniformis,* which frequently differ in accessory genes associated with metabolism, stress responses, and secondary metabolite biosynthesis [53,54].

The most pronounced differences between the two strains were observed in genes involved in nitrogen and carbohydrate metabolism. Although both genomes contained the membrane-bound nitrate reductase complex (narGHIJK), their nitrate assimilation pathways differed substantially: *B. subtilis* RKM-1 possessed the *nasABD* operon, whereas *B. licheniformis* RKM-2 contained *nasC* together with the nitrite reductase genes *nirBCD*. These differences are consistent with the previously reported variability of assimilatory nitrate reduction pathways within the family *Bacillaceae* and may enhance the ecological adaptability of the strains under different nitrogen availability conditions [12,39].

The larger complement of glycoside hydrolases identified in *B. licheniformis* RKM-2, particularly the presence of *celA*, indicates an enhanced potential for the degradation of cellulose-rich plant biomass. Glycoside hydrolases are key enzymes involved in bacterial polysaccharide deconstruction and play an essential role in the degradation of structural plant polymers in natural and engineered microbial ecosystems [46,55]. In contrast, the presence of the *pksA–pksM–pksR* biosynthetic gene cluster in *B. subtilis* RKM-1 suggests the capacity to produce secondary metabolites that may contribute to microbial competition and the suppression of phytopathogenic microorganisms [6,11,48]. Although experimental validation is required to confirm metabolite production, the genomic data indicate that this strain may contribute to the biological stabilization of microbial communities during organic matter transformation.

Overall, the complementary metabolic characteristics identified in the two genomes suggest that a microbial consortium comprising *B. subtilis* RKM-1 and *B. licheniformis* RKM-2 could provide broader functional capabilities than either strain alone. Such a combination may enhance the decomposition of plant residues, improve nutrient recycling, and increase the agronomic value of vermicompost and other organic fertilizers.

From a practical perspective, the application of selected microbial inoculants to large volumes of organic waste requires consideration of several engineering and operational factors. These include the production of sufficient quantities of viable inoculum, homogeneous distribution of microbial cells throughout the composting mass, maintenance of adequate moisture and aeration, interactions and competition with indigenous microbial communities, and the additional costs associated with inoculum preparation and application. In the present pilot-scale experiment, the inoculum was applied by spraying during pile formation followed by thorough mixing, which facilitated its distribution throughout the 300-kg composting piles. At larger scales, inoculum application could be integrated with existing compost-management operations, including mechanical mixing, windrow turning, irrigation systems, or other routine compost-management procedures. The spore-forming capacity and environmental tolerance of *Bacillus* spp. may represent additional advantages for large-scale application because these microorganisms can withstand fluctuations in temperature, moisture, and nutrient availability during composting [56]. Nevertheless, the effectiveness observed under pilot-scale conditions should not be directly extrapolated to industrial systems without further validation.

Importantly, the technical feasibility of microbial augmentation has previously been demonstrated at substantially larger composting scales. Dong et al., for example, evaluated an inoculum containing *B. subtilis* and *Aspergillus niger* during industrial-scale cow-manure composting and reported enhanced thermophilic performance and organic matter transformation [56]. This evidence supports the technical feasibility of microbial augmentation at larger scales, while also indicating that inoculum performance depends on substrate characteristics, pile configuration, aeration strategy, moisture management, and interactions with the indigenous microbiome. Accordingly, further evaluation of RKM-1 and RKM-2 should include larger windrow- or reactor-scale trials together with optimization of inoculum dosage, application strategy, mixing regime, process control, and economic feasibility.

The pilot-scale composting approach used in the present study differs conceptually from standardized OECD/ISO biodegradability test systems. Standardized biodegradation tests are primarily designed to provide reproducible quantitative assessments of degradation and ultimate mineralization under defined and controlled experimental conditions, whereas the present study was designed to evaluate the performance of selected bacterial strains during the composting of a heterogeneous sewage sludge–plant residue matrix under pilot-scale environmental conditions [57]. In the present experiment, temperature dynamics and changes in the C:N ratio were used as process-level indicators of microbial activity and compost stabilization. Standardized approaches, in contrast, may include direct respirometric endpoints such as oxygen consumption or CO_2_ evolution, thereby providing quantitative information on substrate mineralization. Thus, the two approaches should be regarded as complementary rather than directly interchangeable.

Nevertheless, incorporation of standardized respirometric endpoints would strengthen the further validation of the investigated microbial inoculants. In particular, measurement of cumulative CO_2_ evolution would provide a direct quantitative indicator of aerobic carbon mineralization and would complement the temperature and C:N data obtained in the present study. As discussed in the recent review by Strotmann et al. [57], standardized OECD/ISO-based approaches provide important advantages in terms of reproducibility and comparability of biodegradation measurements. Therefore, future studies should combine pilot- or industrial-scale composting trials with controlled CO_2_-evolution or respirometric assays. Such an integrated approach would allow the performance of RKM-1 and RKM-2 to be evaluated both under practically relevant composting conditions and using standardized quantitative biodegradation endpoints.

## 5. Conclusions

The present study provides a comprehensive genomic characterization of two environmental *Bacillus* isolates, *B. subtilis* RKM-1 and *B. licheniformis* RKM-2, using whole-genome sequencing, comparative genomics, phylogenomic analysis, and functional genome annotation. The identified genomic features suggest that the two strains perform distinct yet complementary roles in organic matter decomposition. While *B. licheniformis* RKM -2 appears to be primarily specialized in the degradation of complex carbohydrates and carbon cycling, *B. subtilis* RKM-1 combines the genetic potential for nitrogen assimilation with the capacity for secondary metabolite biosynthesis and phosphorus mobilization. Such functional complementarity is particularly advantageous for the development of microbial inoculants for composting, vermicomposting, and biofertilizer production, where efficient degradation of plant residues requires the coordinated decomposition of carbon-rich substrates, nutrient recycling, and suppression of undesirable microorganisms. More broadly, the strong agreement between comparative genomic analyses and experimental composting data highlights the potential of genome-guided approaches for the selection of microorganisms for environmental biotechnology. Unlike conventional species-based identification, comparative genomics enables the identification of strains with complementary metabolic capabilities, thereby improving the efficiency and stability of organic waste bioconversion processes.

## Figures and Tables

**Table 1:** Extracellular enzymatic activities of *B. subtilis* RKM-1 and *B. licheniformis* RKM-2.

Strain	Cellulase (EI)	Amylase (EI)	Chitinase (EI)
*B. subtilis* RKM-1	5.5 ± 0.79	2.5 ± 0.21	6.9 ± 0.95
*B. licheniformis* RKM-2	8.8 ± 0.29	2.2 ± 0.03	8.5 ± 0.00

Abbreviation: EI—enzymatic index.

**Table 2:** Detailed genome assembly and annotation statistics for strain RKM-1 and RKM-2.

Parameter	RKM-1	RKM-2.
Taxonomic identity	*Bacillus* *subtilis*	*Bacillus* *licheniformis*
Number of reads	1,308,688	1,293,926
Mean read length	295 bp	297 bp
Draft genome size (assembly report)	4,066,570 bp	4,217,124 bp
Genome size (Bakta summary)	4,066,665 bp	4,218,192 bp
GC content	43.7%	46.1%
Total contigs	16	19
Contigs > 1000 bp	14	15
N50	2,094,782 bp	769,660 bp
N90	207,164 bp	210,373 bp
CDS	4070	4315
tRNA	76	81
rRNA	10	11
ncRNA	29	95
Plasmids	0	0
Closest cgSNP neighbor	OH131.1	strain 7712/PD011/PD012
cgSNP distance	86	273

## Data Availability

The raw whole-genome sequencing data generated in this study have been deposited in the NCBI Sequence Read Archive (SRA) under BioProject accession PRJNA1536739. The BioSample accessions are SAMN63657041 for *B. subtilis* RKM-1 and SAMN63657042 for *B. licheniformis* RKM-2, with SRA experiment accessions SRX35457518 and SRX35457519, respectively.
